# Effects of Cannabinoids on Intestinal Motility, Barrier Permeability, and Therapeutic Potential in Gastrointestinal Diseases

**DOI:** 10.3390/ijms25126682

**Published:** 2024-06-18

**Authors:** Kijan Crowley, Łukasz Kiraga, Edyta Miszczuk, Sergiusz Skiba, Joanna Banach, Urszula Latek, Marta Mendel, Magdalena Chłopecka

**Affiliations:** 1 Division of Pharmacology and Toxicology, Department of Preclinical Sciences, Institute of Veterinary Medicine, Warsaw University of Life Sciences—SGGW, Ciszewskiego 8, 02-786 Warsaw, Poland; kijan_crowley@sggw.edu.pl (K.C.); edyta_miszczuk@sggw.edu.pl (E.M.); urszula_latek@sggw.edu.pl (U.L.); marta_mendel@sggw.edu.pl (M.M.); 2 Department of Research and Processing Seed, Institute of Natural Fibers and Medicinal Plants—National Research Institute, Wojska Polskiego 71b, 60-630 Poznan, Poland; joanna.banach@iwnirz.pl

**Keywords:** cannabinoids, cannabidiol, cannabinoid receptors, gastrointestinal motility, gastrointestinal barrier permeability, gut diseases

## Abstract

Cannabinoids and their receptors play a significant role in the regulation of gastrointestinal (GIT) peristalsis and intestinal barrier permeability. This review critically evaluates current knowledge about the mechanisms of action and biological effects of endocannabinoids and phytocannabinoids on GIT functions and the potential therapeutic applications of these compounds. The results of ex vivo and in vivo preclinical data indicate that cannabinoids can both inhibit and stimulate gut peristalsis, depending on various factors. Endocannabinoids affect peristalsis in a cannabinoid (CB) receptor-specific manner; however, there is also an important interaction between them and the transient receptor potential cation channel subfamily V member 1 (TRPV1) system. Phytocannabinoids such as Δ9-tetrahydrocannabinol (THC) and cannabidiol (CBD) impact gut motility mainly through the CB1 receptor. They were also found to improve intestinal barrier integrity, mainly through CB1 receptor stimulation but also via protein kinase A (PKA), mitogen-associated protein kinase (MAPK), and adenylyl cyclase signaling pathways, as well as by influencing the expression of tight junction (TJ) proteins. The anti-inflammatory effects of cannabinoids in GIT disorders are postulated to occur by the lowering of inflammatory factors such as myeloperoxidase (MPO) activity and regulation of cytokine levels. In conclusion, there is a prospect of utilizing cannabinoids as components of therapy for GIT disorders.

## 1. Introduction

The endocannabinoid system (ECS) is a biochemical system engaged in the regulation of numerous physiological and cognitive processes. Functions of this system include the regulation of neurotransmission by interacting with presynaptic receptors in the central nervous system and control of many physiological processes through direct activation of receptors in tissues and organs. This article thoroughly discusses the molecular mechanisms explaining the role of the endocannabinoid system in the gastrointestinal (GI) tract, detailing the proven and alleged involvement of individual components of this system in the regulation of GI motility and intestinal barrier permeability. Our review gives a broad view on the topic discussed, as it also highlights contradictions in the results of the cited studies and indicates areas of knowledge that need confirmation and clarification. By analyzing the molecular relationships between the gastrointestinal endocannabinoid system, the non-cannabinoid receptor pathway, and cannabinoids with well-defined mechanisms of action, the therapeutic potential of individual compounds can be discussed. This focus on phytocannabinoids, which are cannabis-derived bioactive compounds, whose role in medicine is becoming increasingly important, emphasizes their potential to counteract and treat disorders of gastrointestinal motility and intestinal barrier permeability, which are now very common health problems worldwide. However, their application in this context, due to the small number of clinical trials, is so far negligible, which is likely to change as knowledge advances.

## 2. Endocannabinoid System (ECS) 

The ECS is an incredibly important biochemical system involved in numerous vital physiological and cognitive processes. It operates indirectly by regulating the levels and activity of most other neurotransmitters, primarily gamma aminobutyric acid (GABA) and glutamate. In this manner, through immediate feedback, it enhances or diminishes the activity of any system requiring regulation. The ECS controls physiological functions such as maintaining energy balance, ensuring proper blood pressure and body temperature, pre- and postnatal development, suppression of the vomiting reflex, appetite stimulation, pain sensation suppression, and proper inflammatory responses. Regarding gut motility, the ECS affects the digestive system by regulating peristaltic movements and intestinal barrier permeability, as elaborated in this paper. Components of the ECS include cannabinoid receptors, enzymes that regulate the biosynthesis and degradation of cannabinoids, and endocannabinoids [1,2,3].

### 2.1. ECS—Cannabinoid Receptors

Two types of cannabinoid receptors (CBRs) are distinguished: CB1 and CB2. These are metabotropic receptors coupled with Gi/o proteins, making them inhibitory receptors. By inhibiting adenylate cyclase, they decrease the synthesis of cAMP, which is recognized for its role as a secondary messenger interacting with various ion channels. These include inwardly rectifying potassium channels as well as calcium channels that are activated through cAMP-dependent interactions, resulting in a positive influence [4,5]. However, this does not apply to CB2 receptors, which are not coupled with ion channels [6]. Each receptor of this type consists of a single polypeptide chain with seven hydrophobic transmembrane domains that traverse the cell membrane. Ligands bind to it within the hydrophobic region. The difference between them is based on the amino acid structure of the transmembrane peptide and their expression in the organism. The homology of human CBR types is about 44% [7,8]. 

#### 2.1.1. ECS—CB1 Receptors

CB1 receptors exhibit a high degree of conservation across vertebrate species and are also discovered in certain invertebrates. In humans, they are encoded by the *CNR1* gene. Initially cloned from rats, a human CB1 receptor cDNA was subsequently identified. Interestingly, the human CB1 receptor displays one less amino acid in its N-terminus compared to other mammalian species (472 amino acids versus 473 amino acids). The similarities between the rat and human receptors are significant, boasting 93% identity at the nucleic acid level and 97% at the amino acid level. Similarly, the mouse and rat clones share 95% nucleic acid identity (100% amino acid identity), and the mouse and human clones exhibit 90% nucleic acid identity (97% amino acid identity) [8]. It is worth noting that the CB1 receptor stands out as the most abundant G protein-coupled receptor (GPCR) within the mammalian brain [9]. CBRs are found in the spinal cord (where they participate in pain signaling modulation [10]), as well as in peripheral sensory neurons and the autonomic nervous system [11]. In the GIT, cannabinoid type 1 (CB1) receptors are located in neurons of the enteric nervous system (mainly in the myenteric plexus), epithelial cells, and sensory endings of both vagal and spinal neurons. In the ENS circuit, CB1 receptors are located on the excitatory motoneurons, ascending interneurons, and intrinsic primary afferent neurons (IPANs). Activation of CB1 receptors has been demonstrated to regulate multiple functions in the GI tract, encompassing gastric secretion, gastric emptying, and intestinal motility [12,13]. CBRs are primarily located presynaptically and exert their main influence through negative feedback on the synaptic neurotransmission of other neurotransmitters, such as acetylcholine (ACh), glutamate, and GABA [14]. Apart from the nervous system, mild expression can be observed in the liver, spleen, adipose tissue, eye, testes, uterus, and even embryonic cells [15]. 

#### 2.1.2. ECS—CB2 Receptors

The CB2 receptor, which in humans is encoded by the *CNR2* gene, was initially isolated from HL60 cells, a human promyelocytic leukemic cell line [7]. While CB1 receptors are primarily associated with the nervous system, CB2 receptors are mainly found on cells within the immune system and are involved in immunomodulation [4]. However, recent findings indicate that CB2 receptors are also expressed in the brain, by microglial cells and even neurons [8,16]. Their expression primarily occurs in lymphoid organs, including lymph nodes, spleen, thymus, and tonsils, as well as blood cells, mature B lymphocytes and macrophages, and in smaller amounts on mast cells, NK cells, CD4+, and CD8+ lymphocytes. It is also present in the bones, where its expression has been found in bone marrow mesenchymal stem cells [17] as well as osteoclasts [18]. Interestingly, according to some reports, the CB2 receptor was also found in the autonomic nervous system, where it was found in presynaptic membranes, but its function here has not been fully elucidated [19]. This receptor has been associated with diverse regulatory roles, encompassing the control of cell movement, phagocytosis, and primarily, the inhibition of immune cell activity. This immune-suppressive effect results from the reduction in the release of pro-inflammatory cytokines (such as TNF-a, IL-1b, IL-1Ra), the inhibition of cyclooxygenase-2 (COX-2) production, and the suppression of CD40 expression [4]. Regarding the GIT, CB2 receptors are primarily located on immune cells within the gut-associated lymphoid tissue (GALT), which is connected to the modulation of inflammatory responses in the intestines. CB2 receptors are also present on the neurons of ENS and epithelial cells, especially in pathological conditions [20]. Hence, the CB2 receptor presents itself as a promising focus for addressing inflammatory bowel diseases through therapeutic approaches, which is the main subject of this paper. 

Some authors claim that GPR55, GPR119, and GPR18 should be implicated as novel cannabinoid receptors, as they can be agonized by cannabinoids [21,22]. The determination of specific biological functions of these receptors, however, requires further investigation.

**Figure 1:**
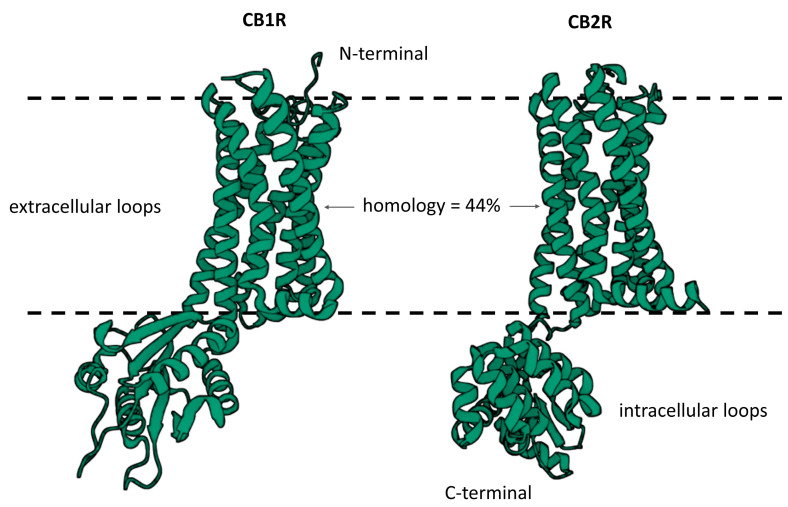
**Three-dimensional visualization of human type 1 and type 2 cannabinoid receptors based on crystallographic data.** CB1 and CB2 receptors are metabotropic receptors coupled with Gi/o proteins. These receptors consist of a single polypeptide chain with seven hydrophobic transmembrane domains traversing the cell membrane. These hydrophobic domains contain binding sites for ligands. Intracellular loops are the site of interaction with the G protein. Homology between CB receptors is approximately 44%. Crystallographic data published by Hua et al. were used to visualize the CB1 receptor [23], and crystallographic data by Li et al. were taken to visualize the structure of the CB2 receptor [24]. Visualization was done in “PDBe-KB 3D viewer online”.

Three-dimensional schematics of human cannabinoid receptor types 1 and 2 are shown in Figure 1.

**Figure 2:**
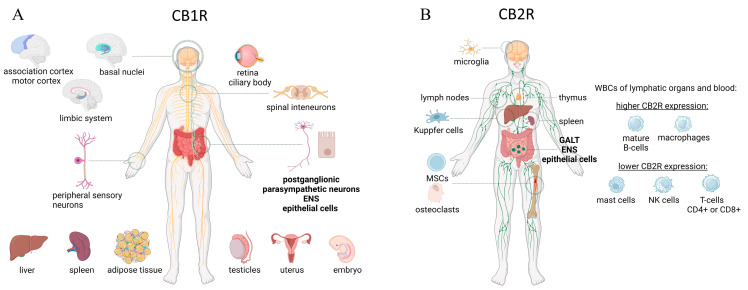
A. Distribution of CB1 (**A**) and CB2 (**B**) receptors in the body. The figure was created with BioRender.com, accessed on 29 April 2024.

The schematics representing distribution of CB1 and CB2 receptors in the body are shown in Figure 2.

#### 2.1.3. ECS—Enzymes Regulating Biosynthesis and Biodegradation of Cannabinoids

The most prominent and well-studied endocannabinoids acting as transmitters in the ECS are anandamide (AEA) and 2-arachidonoylglycerol (2-AG). They follow distinct pathways of biosynthesis and degradation. Anandamide is biosynthesized from N-arachidonoylphosphatidylethanolamine (NAPE). In turn, NAPE arises by transfer of arachidonic acid from arachidonic acid containing phospholipids (i.e., lecithin) to phosphatidylethanolamine through an N-acyltransferase enzyme. Subsequently, involving the enzyme N-arachidonoylphosphatidylethanolamine phospholipase D (NAPE-PLD), from NAPE, phosphatidic acid is removed, which leads to anandamide creation. However, anandamide synthesis from NAPE may also occur via other pathways and includes enzymes such as phospholipase A2, phospholipase C, or ABHD4 enzyme; during transformations mediated by these enzymes, various intermediate compounds are formed from NAPE. The natural occurrence of anandamide is minimal, and it has a brief duration of activity because of the enzymatic action of fatty acid amide hydrolase (FAAH), which breaks it down into free arachidonic acid and ethanolamine [25,26].

Regarding 2-AG, it is synthesized from arachidonic acid-containing diacylglycerol (DAG), which is derived from phosphatidiloinositol or phosphatidic acid (membrane phospholipids) involving the activity of phospholypase C_β_ (PLCβ). DAG is subsequently hydrolyzed by the enzyme diacylglycerol lipase (DAGL), resulting in the formation of 2-AG. Another pathway for its biosynthesis also exists—instead of DAG, with the involvement of the enzyme phospholipase A2 (PLA2), 2-arachidonyl-3-phosphate is produced, which is then transformed into 2-AG through phospholipase C. Similar to anandamide, shortly after its formation, it undergoes biodegradation, facilitated by the enzyme monoacylglycerol lipase (MAGL), leading to the creation of arachidonic acid and glycerol [25,26]. The biological significance of these endocannabinoids is detailed in the subsequent section.

### 2.2. Endocannabinoids

#### 2.2.1. Anandamide

Anandamide was the first endocannabinoid ever discovered (in 1992 by Raphael Mechoulam [27]) and its name comes from ‘ananda’, the Sanskrit word for ‘bliss’. It is an ethanolamide derivative of arachidonic acid. Anandamide features a lengthy lipid chain composed of 20 carbon atoms. Additionally, at one end, it possesses an amino group through which it interacts with cannabinoid receptors. It also has an ethanolamine group that connects the lipid portion with the amino group, acting as a bridge between them [28]. Anandamide is a high-affinity, partial agonist of cannabinoid receptors. Furthermore, it is capable of stimulation of TRPV1, transient receptor potential cation channel subfamily V member 1 (vanilloid receptor 1) [12]. Subsequent studies indicate that anandamide triggers CB1 receptors within the central nervous system as well as localized peripherally, CB2 receptors in peripheral tissues, which encompass immune cells, and TRPV1 receptors throughout various tissues [25]. It serves as the predominant transmitter in the ECS, and all biological functions of this system are contingent upon its activity. Interestingly, anandamide has also been found in certain species of plants and as an alcaloid, and there is a small amount of it in chocolate [29].

#### 2.2.2. Endocannabinoids—2-AG

2-arachidonoylglycerol (2-AG) was the second discovered endocannabinoid, and this was accomplished once again by Mechoulam, in 1995 [27]. It is an ester formed from omega-6 fatty acid, arachidonic acid, and glycerol. Contrary to anandamide, it exhibits the activity of a full CB1 receptor agonist, acting both centrally and peripherally, although its affinity to this receptor subtype is lower than anandamide. Interestingly, 2-AG stands as the most abundant naturally occurring cannabinoid compound within the brain, and findings from electrophysiological investigations propose that 2-AG, rather than anandamide, plays a pivotal role as the primary endocannabinoid in retrograde signaling within the brain [30].

#### 2.2.3. Other Endocannabinoids

The discovery of anandamide and 2-AG was followed by the isolation of other endocannabinoids. These include the noladine ether (ether derivative of 2-AG), virodamin, N-arachidonoyl dopamine, and palmitoylethanolamide (PEA) [31]. Their role in the functioning of the organism seems to be less critical, and deeper investigation is needed to assign them specific biological functions. It is understood that they operate as cannabinoid receptor agonists with either high or low efficacy. Nonetheless, during certain experiments, virodhamine, among them, exhibited characteristics of a CB1 receptor antagonist or inverse agonist [32].

## 3. Phytocannabinoids

Phytocannabinoids are not only found in the cannabis genus but in other plants like *Echinacea purpurea*, *Echinacea angustifolia*, *Echinacea pallida*, *Acmella oleracea*, *Helichrysum umbraculigerum*, the Rhododendron genus, and *Radula marginata*. They are also found in some fungi and liverworts. Phytocannabinoids are meroterpenoids with a resorcinol core and an isoprenyl, alkyl, or aralkyl para-positioned side chain, or an alkyl group usually containing an odd number of carbon atoms [33].

**Figure 3:**
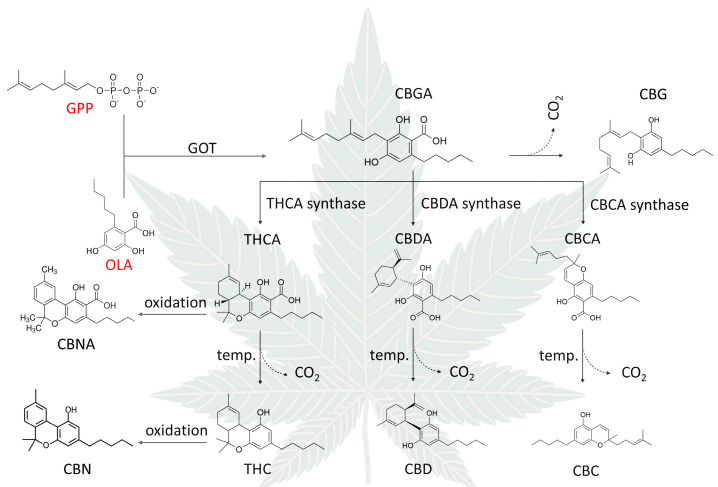
Diagram of phytocannabinoid biosynthesis.

Phytocannabinnoids represent a group of C21 or C22 (for the carboxylated forms) terpenophenolic compounds mainly produced in cannabis. Cannabigerolic acid (CBGA) is the starting point for biosynthesis of the most important cannabinoids. It is formed through the reaction of geranyl pyrophosphate (GPP) with olivetolic acid [34]. This reaction is catalyzed by the enzyme geranylpyrophosphate–olivetolate geranyltransferase (GOT). CBGA is the starting point for biosynthesis of the cannabinoid acids, tetrahydrocannabinolic acid (THCA), cannabidiolic acid (CBDA), and cannabichromenic acid (CBCA), catalyzed by their respective synthases. All cannabinoid acids can be decarboxylated to their active forms through heating or combustion. Tetrahydrocannabinol (THC) and its acid analogue THCA can be nonenzymatically oxidized to cannabinol (CBN) and cannabinolic acid (CBNA), respectively. The detailed pathway of phytocannabinoid biosynthesis is shown in Figure 3.

### 3.1. Phytocannabinoids—THC

THC is a psychoactive cannabinoid found in *Cannabis sativa* subsp. *indica* leaves and flowering plants, and its concentration in these tissues is 0.1-1.6% [35]. (−)-trans-Δ9-THC and (−)-trans-Δ8-THC are the most well-known and studied phytocannabinoids. The unsaturated bond in the cyclohexene ring is located between C-9 and C-10 in the more common dibenzopyran ring numbering system. In (−)-trans-Δ8-THC, the double bond is located between C8 and C9. THC is not soluble in water, and as a lipophilic substance it is distributed to the adipose tissue, liver, lung, spleen, and nervous system. THC exerts its effect by binding to CB1 receptors localized mainly in the nervous system and CB2 receptors found on immune cells [36]. These receptors are coupled with Gi proteins, lowering intracellular cAMP concentration via inhibition of adenylate cyclase. THC is metabolized into 11-hydroxy-Δ9-tetrahydrocannabinol (11-OH-THC) and then into its primary inactive metabolite, 11-nor-9-carboxy-Δ9-tetrahydrocannabinol (THC-COOH), which is glucuronidated. Then, 65% of THC is excreted with the feces and 20% with urine as hydroxylated and carboxylated metabolites [37]. THC’s clinical properties include anti-emetic effects, appetite stimulation, anticonvulsant effects, and analgesia [35]. It is worth noting that this is the only cannabinoid exhibiting psychoactive properties.

### 3.2. Phytocannabinoids—CBD

In contrast to THC, cannabidiol (CBD) is a non-psychoactive substance and does not agonize CB1 and CB2 receptors [38]. CBD is a negative allosteric modulator of the CB1 receptor and has effects on a variety of other receptors and ion channels [39]. CBD has about 6% absorption orally and 11% when inhaled [40]. CBD has shown promise in clinical trials as an anxiolytic, antipsychotic, anticonvulsant, and neuroprotective and anti-inflammatory agent useful in diseases such as Alzheimer’s, drug-resistant epilepsies, and schizophrenia. CBD has very low toxicity in humans and seems to be a safe and effective treatment [4,38].

### 3.3. Other Phytocannabinoids

Other phytocannabinoids, such as cannabichromene (CBC), cannabinol (CBN), cannabigerol (CBG), Cannabidivarin (CBDV), and Δ9-Tetrahydrocannabivarin (THCV), are present in plant tissues in lesser concentrations than CBD and THC and are mostly weak CB1 and CB2 agonists or lack affinity to these receptors. Many of these compounds display affinities to non-cannabinoid receptors and have antimicrobial, anti-inflammatory, and antinociceptive properties [4,41,42,43].

### 3.4. Phytocannabinoid Properties

The properties of the most important phytocannabinoids, including their mechanism of action, are given in Table 1.

**Figure 4:**
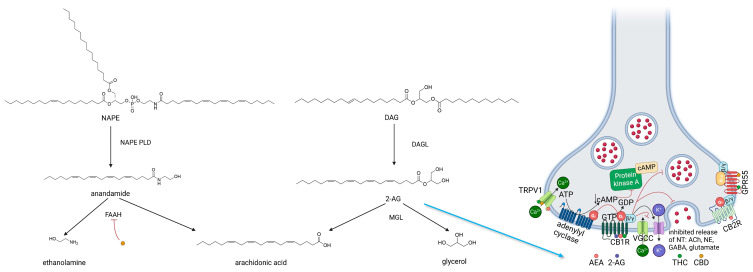
**Diagram of endocannabinoid biosynthesis and mode of action of the endocannabinoid system within a neuron**. AEA and 2-AG activate CB1 receptors, causing a drop in cellular cAMP, inhibition of voltage gated calcium channels (VGCCs), and membrane hyperpolarization by stimulation of potassium channels [49]. This mode of action results the inhibition of neurotransmitter (NT) release into the synaptic cleft. AEA may also act by activating CB2 receptors and via the non-cannabinoid pathway using transient receptor potential cation channel subfamily V member 1 (TRPV1) [50] and G protein-coupled receptor 55 (GPR55) [51]. There is evidence that phytocannabinoids act by the CB receptors and TRP channels, although many data indicate that other mechanisms are also involved [52]. The figure was created with BioRender.com, accessed on 29 April 2024.

The scheme of endocannabinoid biosynthesis, the mechanism of receptor action of endocannabinoids, and the most important phytocannabinoids are shown in Figure 4.

## 4. Effects of Cannabinoids on Gastrointestinal Motility

Endocannabinoids, including AEA, 2-AG, and endocannabinoid-like compounds, have been extensively studied for their effects on gastrointestinal (GI) motility. Additionally, phytocannabinoids like THC, CBD, CBC, and CBN have also garnered significant interest for their potential therapeutic effects on GI contractility disorders. Both ex vivo and in vivo studies have elucidated their roles in modulating contractile responses, neurotransmitter release, and receptor interactions, particularly through cannabinoid receptors CB1 and CB2, as well as other pathways. This section explores the impacts of cannabinoids on GI function through various models, detailing their receptor-mediated mechanisms and therapeutic applications.

### 4.1. Endocannabinoids

#### 4.1.1. Effects of AEA and 2-AG on Gastrointestinal Motility

In ex vivo studies in mice, anandamide reduced spontaneous contractility of ileal longitudinal muscle strips via CB1 [53] and electrically evoked contractions in colon preparations via CB1 [54]. In guinea pig small intestine circular muscle preparations, anandamide reduced cholinergic and non-adrenergic-non-cholinergic (NANC) contractility (induced via electrical field stimulation) via CB1, without altering contractile response to exogenous substance P (SP) and acetylcholine (ACh). It also did not interact with L-NAME, naloxone, or phentolamine [55]. In human colonic longitudinal and circular muscle, anandamide and 2-AG inhibited the contractile response to ACh but did not inhibit the response to KCl, nor did they alter basal muscle tension. This effect on ACh response was not reversed by the CB1 receptor antagonist AM251 or the CB2 receptor antagonist JTE907, suggesting involvement of a non-CB1, non-CB2 pathway. Abolishing endocannabinoid breakdown via the FAAH inhibitor arachidonoyl trifluoromethyl ketone reduced ACh response on its own but did not modify the suppressive effect of anandamide [56]. In rat ileal preparations, anandamide reduced the twitch response to ACh via CB1, by affecting neuro-neuronal and neuro-muscular neurotransmission as well as reducing excitatory junction potentials (EJPs) and inhibitory junction potentials (IJPs), while leaving intestinal slow waves unaffected [57]. An in vivo study demonstrated that anandamide reduced GIT motility and improved postprandial glycemia in mice [58].

Conversely, in guinea pigs, there are reports of anandamide stimulating intestinal contractions. Anandamide was found to increase basal ACh release and muscle tone in isolated guinea pig proximal small intestine myenteric plexus longitudinal muscle strips, an effect inhibited by the vanilloid receptor antagonist capsazepine and a combined blockade of NK1 and NK3 receptors. This was not, however, blocked by the CB1 or CB2 receptor antagonists [50]. This suggests that anandamide exerts its stimulatory effect on basal ACh release via TRPV1 receptor stimulation. In this study, anandamide also inhibited electrically evoked ACh release and contractions, whose effect was not blocked by capsazepine, combined NK1 and NK3 receptor blockade, or CB2 blockade, but was reduced by the CB1 receptor antagonist rimonabant. This inhibition by rimonabant of anandamide’s effect on evoked ACh release was much less potent than rimonabant’s inhibition of the CP55,940 effect on evoked ACh release. These results suggest that anandamide’s inhibition of evoked ACh release is mediated via a receptor other than CB1 or CB2 [50]. In another study, anandamide and 2-AG were observed to contract guinea pig distal colon longitudinal muscle strips by stimulating myenteric cholinergic neurons independently of cannabinoid or vanilloid receptors. This was inhibited by the lipoxygenase inhibitor nordihydroguaiaretic acid, suggesting that lipoxygenase metabolites may mediate these contractions [52]. This would seem to be supported by a later study, in which anandamide caused guinea pig ileal segments to contract, an effect that was strongly inhibited by indomethacin (a nonselective cyclooxygenase inhibitor), tetrodotoxin, or a combination of atropine and an NK1 receptor antagonist. The contractions were weakly reduced by atropine alone and partly diminished by the FAAH inhibitor URB 597. Pre-treatment with capsaicin, a TRPV1 receptor antagonist, hexamethonium, and CB1 and CB2 receptor antagonists all failed to influence the anandamide-induced contraction. Taken together, this suggests that anandamide’s contractile properties are exerted via cyclooxygenase metabolites but do not involve TRPV1 receptors or capsaicin-sensitive neurons, and that the final mediators for the response are ACh and tachykinin peptides [59].

There have been several studies investigating the relationship between anandamide and pathological states of the intestine. Anandamide levels in the whole small intestine were found not to differ between mice with inflammation induced by croton oil and control animals. There was, however, increased activity of FAAH, the enzyme responsible for anandamide breakdown, as well as increased CB1 receptor expression [60]. A later study by the same author, using the same model, analyzed anandamide concentrations in the individual portions of the small intestine, finding that anandamide levels were significantly lowered in the jejunum, but not the duodenum or ileum, of croton oil-treated mice, as compared to the control group. The study also found upregulation of CB1 receptors in the jejunum, and upregulation of TRPA1 receptors and downregulation of CB2 receptors in the entire small intestine [61]. Another study demonstrated an increase in murine whole small intestine anandamide (but not 2-AG or PEA) levels in a model of acetic acid-induced paralytic ileus. Additionally, overexpression of CB1 receptors was observed. Paralytic ileus was alleviated by the CB1 receptor antagonist SR141716A and exacerbated by the anandamide uptake inhibitor VDM11. No significant changes in FAAH activity were observed, as compared to controls, implying that the rise in anandamide concentration was not due to a reduction in inactivation. Taken together, this suggests a causative role for anandamide in paralytic ileus [62]. 

One study, investigating changes in GIT motility and tissue endocannabinoid levels due to obesity, used both rat and mice models. In mice with high-fat diet-induced obesity, baseline intestinal transit was increased, whereas small intestinal anandamide levels were decreased and 2-AG was increased. In addition, overnight fasting increased endocannabinoid levels in the duodenum of both Zucker and lean rat strains. Endocannabinoid dysregulation, in the form of reduced anandamide levels, may be responsible for increased intestinal transit in mice with diet-induced obesity [63]. 

In guinea pig myenteric plexus longitudinal muscle preparations, anandamide at lower concentrations was shown to have an inhibitory effect on ethylenediamine-induced GABA release, decreasing the inhibition of contractions by ethylenediamine. At higher concentration, however, anandamide increased contractile inhibition by ethylenediamine, an effect that was eliminated by the TRPV1 antagonist capsazepine in combination with the GABA(B) antagonist CGP54626A. This suggests that anandamide has an inhibitory effect on electrically induced contractions independently of GABA and CB receptors, via TRPV1. Anandamide also inhibited electrically induced contractions on its own via CB1 [64]. Similarly, anandamide was observed to inhibit electrically induced adenosine release in the myenteric plexus longitudinal muscle of the guinea pig ileum [65].

In another study, anandamide inhibited the contractile response to brushstroke stimulation in three-compartment rat colon preparations (orad motor, central sensory, and caudad motor compartments). Anandamide decreased SP release and ascending contraction in the orad motor compartment, VIP release and descending relaxation in the caudad motor compartment, and release of the sensory transmitter CGRP in the central sensory compartment. The reverse was true for the CB1 antagonist AM-251. This would suggest that the inhibition of peristalsis by cannabinoids is caused by inhibition of both excitatory cholinergic and inhibitory VIPergic motor neurons, as well as sensory neurons initiating the peristaltic reflex [66].

#### 4.1.2. Effects of Endocannabinoid-like Compounds on Gastrointestinal Motility

Naturally occurring endocannabinoid-like compounds palmitoylethanolamide (PEA), oleoylethanolamide (OEA), and oleamide (OA) were demonstrated to have an inhibitory effect on the upper GIT motility of mice. PEA inhibited upper GIT motility in healthy mice [67] and in a model of chronic GI inflammation induced by croton oil administration, where this inhibition was independent of CB1, CB2, opioid, and alpha-2 adrenergic receptors, and was also not blocked by the NO synthase inhibitor L-NAME. In the same study, PEA levels in the small intestine of croton oil-treated mice were significantly lower than in control animals [68]. OEA inhibited small intestinal transit in mice [67,69] but did not slow whole gut transit or colonic propulsion. OEA worked independently of CB1, CB2, PPARα, and TRPV1 and was not blocked by the GLP-1 antagonist exendin-3(9-39) amide [69]. The same study found that OEA blocked in vivo stress-induced hypermotility at dosages that had no effect on physiological transit [69]. OEA, but not PEA, slowed gastric emptying independently of CB1, CB2, and PPARα receptors and was present at higher levels in the stomachs of mice fed a high-fat diet [70]. OA slowed small intestinal transit in mice [67].

#### 4.1.3. Summary of Endocannabinoid Influence on Gastrointestinal Motility

**Figure 5:**
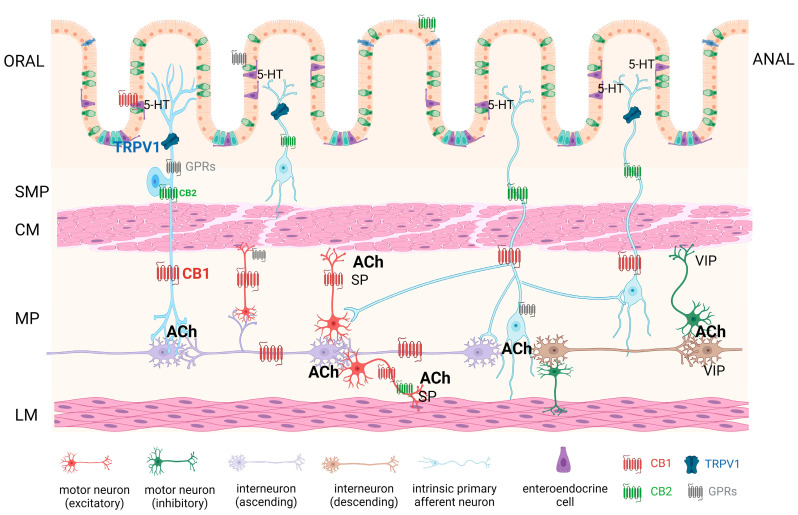
Distribution of receptors in the enteric nervous system (ENS) involved in regulating gastrointestinal motility by cannabinoids. AEA and phytocannabinoids influence cannabinoid receptors 1 (CB1) located on enteric neurons, inhibiting the release of acetylcholine (ACh) and muscle contractility [8,71]. The role of CB2 receptors in the regulation of motility is significantly smaller and seems to be important in pathophysiological states such as inflammation [6]. AEA can influence gut contractility via a non-cannabinoid receptor pathway, through the transient receptor potential cation channel subfamily V member 1 (TRPV1) [50], located on the enteric neurons, and G protein-coupled receptor 55 (GPR55) [51], as a putative endocannabinoid receptor. CM—circular muscles, LM—longitudinal muscles, MP—myenteric plexus, SMP—submucosal plexus, VIP—vasoactive intestinal peptide, SP—substance P. The figure was created with BioRender.com, accessed on 29 April 2024.

In summary, endocannabinoids and endocannabinoid-like compounds seem to exert mainly inhibitory effects on GIT contractility. In contrast, several studies have observed the opposite to be true. The majority of studies report activity at the CB1 and TRPV1 receptors for these compounds; however, some papers have found endocannabinoids to influence GIT motility via pathways that were not able to be fully classified, some pointing to possible involvement of lipoxygenase metabolites. A few research papers have also linked GIT endocannabinoid dysregulation to pathological states of the intestine, although the relationship is not yet clear. A summary of endocannabinoid influence on gastrointestinal motility is given in Table 2 and schematically shown in Figure 5.

### 4.2. Effects of Phytocannabinoids on Gastrointestinal Motility

#### 4.2.1. THC

Data concerning the impact of phytocannabinoids on the GIT first appeared quite a long time ago. THC slowed gastrointestinal transit of a charcoal meal in live mice independently of opioid receptors [72,73] and also inhibited the contractile response of the isolated guinea pig ileum to electrical and 5-HT stimulation. It did not, however, inhibit the reaction to ACh and histamine, which act directly at the level of smooth muscle, suggesting that THC exerts its effect presynaptically [74]. Inhibition of contractility in guinea pig small intestine myenteric plexus preparations by THC was achieved via CB1 receptor stimulation, independently of opioid receptors [75,76]. Low doses of THC were found to counteract the inhibitory effect of prostaglandin E1 (PGE1) on GI motility in mice, as measured by charcoal meal transit time, but at higher doses was itself inhibitory [77]. In another study, inhibition of electrically induced contractility via CB1 agonism was observed in rat and guinea pig ileal myenteric plexus longitudinal muscle preparations; however, the inhibition of rebound contractions was not mediated via CB1, CB2, or TRPV1 [78].

#### 4.2.2. Effect of CBD on Gastrointestinal Motility

CBD was not observed to influence electrically induced contractility in the isolated guinea pig ileum [74]; however, in combination with THC, it inhibited the speed of transit of a charcoal meal in mice to a greater degree than THC on its own [72]. In another study, CBD normalized in vivo hypermotility induced by croton oil in mice, as measured by fluorescent marker transit speed; however, it did not influence motility in the control group. CBD did not inhibit motility in vivo in animals treated with an FAAH inhibitor. In the same study, CBD inhibited contractility induced by ACh and PGF2a in both healthy and croton-treated ileal segments in vitro, but did not inhibit spontaneous contractility [79]. A study by Cluny et al. from 2011 found that both cannabidiol and cannabidiolic acid (CBDA) caused a drop in resting tension of isolated intestinal segments of the Asian house shrew. They also caused a reduction in the magnitude of contractions caused by carbachol administration and reduced the tension of segments pre-contracted by KCl. These effects were not blocked by CB1 or CB2 antagonists, nor were they blocked by tetrodotoxin (TTX), which might suggest that the effect is not neuronally mediated. CBD and CBDA also inhibited electrically induced intestinal contractions [80]. One study showed significant interaction between CBD and steroidal and nonsteroidal anti-inflammatory drugs ex vivo. CBD applied as a sole agent inhibited ACh-induced motility in isolated rat colon preparations. This effect of CBD on contractility was significantly diminished by co-administration with dexamethasone. In contrast, co-administration with diclofenac caused a far greater inhibition of ACh-induced contractility than either substance applied individually. A possible mechanistic explanation of these results is that the nonsteroidal anti-inflammatory drug diclofenac caused an accumulation of arachidonic acid via cyclooxygenase pathway inhibition, thus providing more arachidonic acid for the synthesis of endogenous cannabinoids. The attenuation of the effect of CBD by dexamethasone may be explained by the inverse mechanism—dexamethasone diminishes arachidonic acid availability via phospholipase A2 inhibition. Such interactions are noteworthy, due to the high probability of these two classes of drugs being used in parallel with cannabinoid therapy [81].

In a study using both in vivo and ex vivo models of TNBS-induced colitis in rats, pretreatment with CBD caused a reduction in TNBS-induced upper GIT hypermotility in vivo and reversed the enhancement of electrically induced contractility caused by TNBS administration in isolated colonic muscle strips ex vivo [82].

#### 4.2.3. Effects of Other Phytocannabinoids on Gastrointestinal Motility

The results of a study investigating CBC showed that this phytocannabinoid normalized in vivo inflammatory hypermotility in mice treated with croton oil but did not have any effect on motility in control animals. In this same study, CBC preferentially inhibited electrically stimulated contractions versus ACh-induced contractions of the intestine in vitro. The inhibitory effect of CBC on ACh-induced contractions was diminished by verapamil, suggesting that CBC exerts this effect through mechanisms involving L-type Ca^2+^ channels. The inhibitory effect of CBC on electrically induced contractions was found to be attenuated by the N-type Ca^2+^ channel blocker omega-conotoxin, suggesting the involvement of these channels in CBC’s mechanism of action. Both in in vivo and in vitro conditions, the effect of CBC on contractility was not attenuated by CB1, CB2, or TRPA1 receptor antagonists [61].

Another phytocannabinoid, CBN, was found to inhibit the contractile response of guinea pig intestinal tissue to electrical stimulation in vitro, but this inhibition was not affected by the CB1 antagonist rimonabant [76], p. 199. In contrast, an earlier study did not detect any effect of CBN on guinea pig in vitro intestinal twitch response to ACh, histamine, or 5-HT [74]. The results of a later study, however, showed inhibition of in vivo colonic propulsion in mice via CB1, but not CB2, receptor activation [83].

#### 4.2.4. Summary of Phytocannabinoid Influence on Gastrointestinal Motility

Summing up, phytocannabinoids show a strong ability to inhibit spontaneous and induced GIT contractions, which appears to be mediated via CB1; however, it is uncertain whether this occurs directly, through negative allosteric modulation, or indirectly, by inhibiting anandamide reuptake. A summary of phytocannabinoid influence on gastrointestinal motility is given in Table 3.

## 5. Effects of Cannabinoids on Intestinal Barrier Permeability

The endocannabinoid system is known to play an important role in modulating intestinal permeability. In rats, centrally administered oxytocin decreases LPS-induced intestinal hyperpermeability partly via CB1 signaling [84]. Changes in intestinal permeability associated with cellular senescence have also been shown to be related to decreased cellular CB1 expression [85].

### 5.1. Effects of Endocannabinoids on Intestinal Barrier Permeability

#### 5.1.1. Anandamide and 2-AG

There have been several studies showing the significant effects of endocannabinoids on intestinal permeability, mostly using Caco-2 cell culture models. Endocannabinoids differ in their impact on permeability, depending on which substance is used and whether it is applied apically or basolaterally to cell cultures.

Anandamide and 2-AG applied apically to Caco-2 cell cultures caused a further decrease in transendothelial electrical resistance (TEER) to that caused by EDTA. When administered basolaterally, however, both endocannabinoids caused a concentration-dependent recovery of TEER values after the drop evoked by the application of EDTA. This was inhibited by the CB1 receptor antagonist AM251 and, in the case of anandamide, additionally by the TRPV1 antagonist capsazepine. Both cannabinoids also caused downregulation of mRNA for the TJ protein claudin-1, which was inhibited by AM251 [86]. A similar effect occurred in in Caco-2 cultures treated with IFNg and TNFa, where apical application of anandamide and 2-AG worsened hyperpermeability as measured by TEER and fluorescein isothiocyanate–dextran (FD4) flux. The CB1 receptor antagonist AM251 inhibited the exacerbation of hyperpermeability induced by the two endocannabinoids. Furthermore, anandamide given alongside an FAAH inhibitor lowered TEER, as did the FAAH inhibitor applied at the same time as the cytokines. Analogously, 2-AG alongside a monoacylglycerol lipase (MAGL) inhibitor lowered TEER to a greater degree than 2-AG alone, as did the MAGL inhibitor administered at the same time as the cytokines. The effects of both FAAH and MAGL inhibitors on TEER were inhibited by AM251, implying that their influence was due to an increase in endocannabinoid concentrations caused by inhibition of their breakdown. This suggests that locally produced cannabinoids play a part in cytokine-mediated intestinal permeability changes [87]. 

When administered apically to the Caco-2 monolayer, the FAAH inhibitor URB597 and the MAGL inhibitor JZL184 caused a concentration-dependent drop in TEER, which was reversed by AM251, a CB1 receptor antagonist. When URB597 was applied basolaterally, the same phenomenon occurred, this time inhibited by AM251 and the TRPV1 antagonist capsazepine. In contrast, JZL184 applied basolaterally caused the opposite, an increase in TEER values, whose effect was also inhibited by AM251. 2-AG alone applied basolaterally also caused an increase in resistance. Both URB597 and JZL184 caused a further decrease in TEER in Caco-2 cells exposed to inflammatory cytokines, as well as preventing recovery of TEER in cell cultures exposed to hypoxia. No effects on TEER were found when URB597 or JZL184 were administered apically or basolaterally in CB1 knockdown Caco-2 cells. The lack of contribution of TRPV1 in CB1 knockdown cultures may suggest that the ability of TRPV1 to affect permeability is dependent on activation of CB1 receptors [88]. 

Mice fed a high-fat, high-sucrose diet for 8 weeks experienced elevations in FD4 flux across the intestinal barrier, as compared to mice fed a standard diet. These changes in permeability were accompanied by a drop in the content of 2-AG and other related monoacylglycerols OEA, 2-docosahexaenoyl-sn-glycerol (2-DG), and 2-linoleoylglycerol (2-LG) in the epithelium of the large intestine, as well as a significant decrease in the activity of monoacylglycerol biosynthetic enzymes. Additionally, this high-fat, high-sucrose diet impacted permeability as measured by increased FD4 flux much more strongly in mice lacking intestinal CB1 receptors than in wild-type mice, implying a protective role for 2-AG in intestinal permeability in diet-induced obesity [89].

#### 5.1.2. Effects of Endocannabinoid-like Compounds on Intestinal Barrier Permeability

A wide variety of endocannabinoid-like compounds have been isolated and studied over the years. Studies examining their effect on intestinal permeability have mostly used the Caco-2 cell culture model, with one study including human in vivo experiments. Endocannabinoid-like compounds differ in their effect on intestinal permeability, as well as in the receptors via which they effect these changes. When applied to the apical membrane of Caco-2 cells, OEA caused an increase in TEER, and a decrease in TEER when given basolaterally. The effect on the apical membrane was inhibited by the TRPV1 antagonist capsazepine, and the effect of basolateral application was inhibited by the PPARα antagonist GW6471. OEA given apically at the same time as IFNg and TNFa prevented the development of cytokine-induced hyperpermeability and also recovered TEER values when given 24 h after the cytokines. This was inhibited by capsazepine. Basolateral application of OEA at the same time points exacerbated cytokine-induced permeability. This was inhibited by GW6471, but not capsazepine. PEA increased TEER when introduced both apically and basolaterally, and was inhibited by GW6471 at both locations. PEA applied apically had no effect on cytokine-induced changes in permeability; however, when administered basolaterally, it prevented hyperpermeability when co-applied with cytokines and caused recovery of TEER when given 24 h later. This effect was inhibited by GW6471. Both OEA and PEA reduced membrane expression of aquaporin 3 (AQP3) and aquaporin 4 (AQP4) and led to a concentration-dependent increase in fluorescence, indicating potassium channel activation [90]. In another study, N-arachidonoyl dopamine (NADA), OEA, and OA, administered apically, mitigated hypoxia-induced TEER decrease and sped up recovery. Applied basolaterally, the same was true for NADA, OA, NE, and PEA, but OEA had the opposite effect. Apically, NADA, OA, and OEA exerted their effects through TRPV1 activation. When introduced basolaterally, NADA worked via TRPV1, and OA worked via TRPV1 and CB1. NE and PEA worked via PPARα. OEA worsened permeability via PPARα activation [91]. PEA, when applied basolaterally, reduced both FD4 and FD10 flux across Caco-2 membranes in a state of cytokine-induced hyperpermeability. The effect of PEA in reducing dextran flux was inhibited by PPARα antagonism as well as by inhibition of protein kinase A (PKA), mitogen-associated protein kinase (MAPK), and adenylyl cyclase signaling pathways. In cell cultures, PEA also prevented the cytokine-induced drop in TRPV1 mRNA and the cytokine-induced rise in AQP3 gene expression. Alone, PEA increased AQP4 gene expression in Caco-2 cells. In both normal and inflamed human colonic tissue, PEA caused a drop in claudin-3 mRNA levels. In human subjects given lactulose and mannitol + PEA and aspirin, PEA lowered the urinary lactulose-to-mannitol ratio, as compared to the group given lactulose and mannitol + placebo. Lactulose is not absorbed in the healthy gut, but is passively absorbed in inflammatory states. Aspirin increases the urinary lactulose-to-mannitol ratio, which is hypothesized to happen via COX inhibition. This implies that PEA can reduce intestinal permeability in humans in vivo [92]

#### 5.1.3. Summary of Endocannabinoid Influence on Intestinal Barrier Permeability

In summary, the effect of endocannabinoids on intestinal permeability varies widely depending on the compound used and whether it is applied apically or basolaterally to the Caco-2 monolayer. The impact on permeability is mainly achieved via CB1 and TRPV1, but also PPARα activation. Endocannabinoids also caused changes in the synthesis of certain cellular membrane proteins, which play a role in intestinal barrier integrity. A summary of the effects of endocannabinoids on gastrointestinal permeability is shown in Table 4.

**Figure 6:**
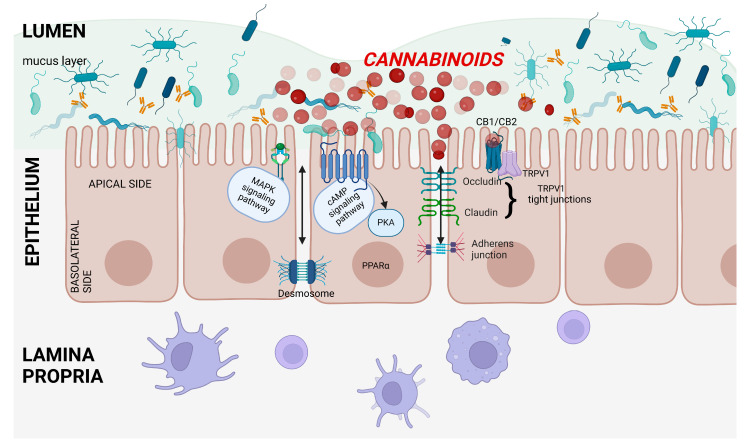
**Intestinal barrier and elements involved in the influence of cannabinoids on its permeability.** The effect of cannabinoids on intestinal permeability is achieved mainly through the CB1, TRPV1, MAPK, and adenylate cyclase signaling pathways, but also through the activation of PPARα. Cannabinoids may also cause changes in the synthesis of tight junction proteins (zonula occludens, claudin, occludin), which play a significant role in regulating the integrity of the intestinal barrier. The figure was created with BioRender.com, accessed on 29 April 2024.

A schematic of the intestinal barrier, including the influence of the endocannabinoid system on the barrier, is shown in Figure 6.

### 5.2. Effects of Phytocannabinoids on Intestinal Barrier Permeability

THC and CBD are the two phytocannabinoids with the largest number of studies describing their impact on intestinal permeability. 

In Caco-2 cell lines, THC and CBD sped up recovery of normal permeability, as measured by TEER, after disruption by EDTA. This was the case with both apical and basolateral administration and was mediated via CB1 receptors. After treatment with THC or CBD, there was significant upregulation of mRNA expression for the TJ protein ZO-1. This effect was also abolished by the CB1 receptor antagonist AM251 [86]. Apical incubation of Caco-2 cells with THC or CBD for 24 (but not 48) h after exposure to IFNg and TNFa accelerated the recovery of normal permeability (as measured by TEER) after the increase in permeability induced by cytokines. Administration of THC or CBD apically at the same time as IFNg and TNFa caused complete inhibition of the cytokine-induced fall in TEER and prevented increased FD4 flux. This effect was inhibited by the CB1 antagonist AM251, but not by CB2, GPR18, GPR55, TRPV1, PPARα, or PPARγ antagonists. Interestingly, the CB1 antagonist AM251 also significantly decreased the fall in TEER when co-administered with the cytokines but had no effect when given 24 h after IFNg and TNFa [87]. A study found no difference between synthetic and plant-derived CBD in their effect on changes in intestinal permeability caused by inflammatory cytokines. Both types of CBD facilitated the return to baseline TEER values in Caco-2 cell monolayers incubated with TNFa and IFNg via CB1 [93].

THC decreased permeability in Caco-2 cell cultures with TEER decreases due to transfection with the inflammation-induced microRNA molecules miR-130a and miR-212. This effect was due to upregulation of PPARγ receptor expression induced by THC [94].

CBD inhibited cytotoxicity and apoptosis induced by *Clostridium difficile* toxin A (TcdA) in Caco-2 cell lines and reversed the decreased gene expression for the TJ proteins ZO-1 and occludin. The above protective effects were mediated by CB1 receptors [95]. In vitro, CBD decreased FD4 and FD10 flux in Caco-2 cell cultures exposed to IFNg and TNFa. This effect was blocked by CB1 antagonism, as well as antagonism of PKA, MAPK, and adenylyl cyclase signaling. CBD also decreased AQP3 gene expression and increased TRPV1 expression, which were respectively increased and decreased due to inflammation [92].

Another in vitro study conducted on Caco-2 cell lines found that CBD did not significantly reverse cytokine-induced hyperpermeability [96]. In contrast, a later study showed a significant protective effect of CBD on Caco-2 membrane permeability exposed to inflammatory and oxidative insults. Pretreatment of membranes with CBD ameliorated the drop in TEER induced by exposure to hydrogen peroxide, and was able to almost entirely prevent the fall in TEER caused by incubation of membranes with INFγ and TNFα. In addition, CBD was able to prevent changes in tight junction morphology caused by INFγ and TNFα administration, as observed by confocal microscopy [97].

In human colonic tissue preparations, CBD counteracted the inflammation-induced decrease in claudin-5 mRNA. In the same study, healthy humans given mannitol, lactulose, and high-dose aspirin experienced a decrease in the ratio of lactulose to mannitol in the urine after treatment with CBD, as compared to the group not given CBD. CBD lowering the urinary lactulose-to-mannitol ratio suggests an in vivo reduction in intestinal permeability [92].

In a murine model of colitis induced with dextran sulphate sodium (DSS), CBD alone was not effective at protecting the gastrointestinal barrier from DSS-induced increases in permeability. However, when CBD was co-administered with fish oil (at ineffective doses, when used alone), the two substances were able to significantly ameliorate the rise in intestinal permeability caused by DSS, as measured by FD4 flux assay [98]. The results of a later study, using a similar model, but substituting DSS for 2,4-dinitrobenzenesulfonic acid (DNBS), showed that CBG administered orally, but not CBD, attenuated the rise in intestinal permeability caused by DNBS administration. The addition of fish oil (at a dose not effective alone) potentiated oral CBD, enabling it to ameliorate the effect of DNBS on permeability, as measured by FD4 flux [99].

#### Summary of Phytocannabinoid Influence on Intestinal Permeability

**Figure 7:**
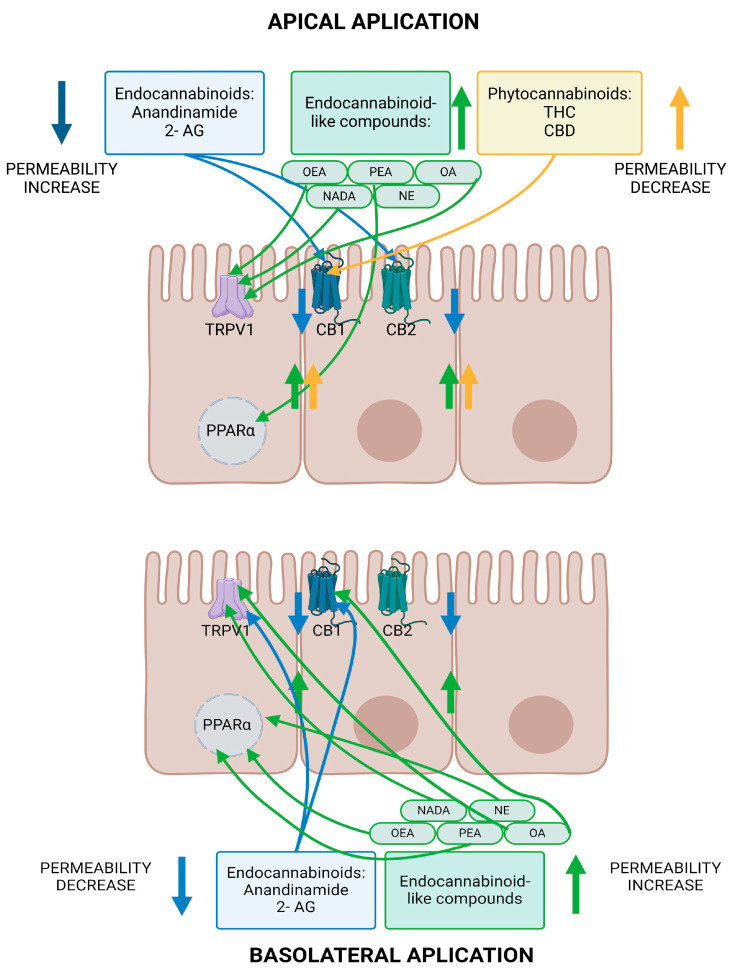
Effect of different groups of cannabinoids on intestinal permeability after apical and basolateral administration to Caco-2 cell culture. Apically administration of endocannabinoids and endocannabinoid-like compounds cause an increase in permeability mediated primarily by CB1, CB2, and TRPV1 receptors and through the PPARα signaling pathway, while when they are administered basolaterally, cause a decrease in permeability via CB1 and TRPV1 receptors and also the PPARα signaling pathway. Phytocannabinoids have been found to improve the integrity of intestinal barrier by influencing CB1 receptors. The figure was created with BioRender.com, accessed on 29 April 2024.

The phytocannabinoids examined above were found to improve intestinal barrier integrity, mainly through CB1 receptor stimulation, but also via PKA, MAPK, and adenylyl cyclase signaling pathways. In one study, intestinal permeability was decreased via PPARγ upregulation. Phytocannabinoids also caused changes in the synthesis of proteins involved in paracellular transport and the intestinal barrier. A summary of phytocannabinoid influence on gastrointestinal permeability is given in Table 5, and a schematic presentation of the effects of different groups of cannabinoids on intestinal permeability for the example of Caco-2 cells is shown in Figure 7.

## 6. Therapeutic Potential of Cannabinoids in Gut Diseases

Recent studies suggest a strong link between changes in endocannabinoid levels and gastrointestinal (GI) diseases like irritable bowel syndrome (IBS) and Crohn’s disease [100]. Elevated endocannabinoid levels and increased CB receptor expression in these conditions raise the possibility of their diagnostic and therapeutic use. Phytocannabinoids, especially THC and CBD, show promise in treating various GI disorders; however, conflicting results in some clinical studies on CBD’s effects underscore the need for further research. This section provides a glimpse into the evolving field of cannabinoid research within the realm of gastrointestinal health, emphasizing both the progress made and the avenues for future exploration.

### 6.1. Endocannabinoids

#### 6.1.1. Endocannabinoids and Diarrhea

An important point to consider in humans is that a single nucleotide polymorphism in the FAAH gene may downregulate its expression. Therefore, any treatment involving the possible use of this enzyme could depend on the individual susceptibility of each patient. A relationship between the genotypic variation associated with the FAAH gene and the functional GIT disorder-prone phenotype has been noted [101]. It is also worth noting that both reduction of pain perception and alleviation of hypermotility and diarrhea were not possible in FAAH knockout mice [102]. The use of aFAAH inhibitor (PF-3845) in in vivo experiments in mice resulted in inhibition of passage in the GIT, both after central (i.c.v.) and peripheral (i.p.) administration. This could be successfully reversed using a CB1 antagonist, bringing the transit speed back to the baseline values or even lower. These effects also occurred in tests using mouse models of GIT hypermotility and diarrhea [102]. The same study also tested whether FAAH inhibition would affect nociception. PF-3845 reduced pain-related behaviors in mice. Only the central effect could be reversed using a CB1 antagonist, and the antinociceptive effect was partially achieved already at very low doses but only after peripheral administration. The study suggested the relevance and validity of further research into the possibility of using FAAH blockers in the treatment of diseases such as IBS with predominant diarrhea (IBS-D). 

In mice, there was an increase in anandamide (but not 2-AG or PEA) levels in the small intestine after oral administration of cholera toxin (CT). Additionally, there was increased expression of CB1 receptors in the intestine. No difference in FAAH activity was observed between animals administered CT and control. Fluid secretion was prevented by administration of the anandamide uptake inhibitor VDM11, but worsened by the CB1 receptor antagonist SR141716A. This suggests that increased CB1 stimulation via increased production of anandamide and upregulation of CB1 receptors exerts a protective effect against cholera toxin-induced hypersecretion [103].

#### 6.1.2. Endocannabinoids and Intestinal Inflammation

Endocannabinoid concentrations and levels of CB1R expression may act as an indicator for celiac disease. This was suggested by a study of thirteen celiac patients in the phase of villous atrophy, seven in complete remission, and a control group of eleven who had never experienced the disease. Anandamide and PEA concentrations in duodenal biopsies from the study group with active disease were remarkably elevated compared to the non-celiac control group. An increase in the concentration of 2-AG was also noted, but not as significant as in the case of the two previous compounds. In this case, the results also turned out to be highly variable between individuals. One of the most significant and important observations in this study was that after switching celiac patients to a gluten-free diet and reduction in villous atrophy, more than half of the patients (7 out of 14) had their anandamide levels return to those found in non-celiac patients. PEA concentrations remained at the same level before and after dietary treatment. Patients in the active phase of the disease had increased CB1R immunofluorescence compared to the results from non-celiac patients and those in remission. This means that the increased expression of CB1R, the elevated concentrations of endocannabinoids and PEA, as well as their changes could serve as indicators of the course of the disease and treatment. Moreover, this study reinforced the idea that endocannabinoids most likely play a protective and homeostatic role during intestinal inflammations of different types, including celiac disease [104]. 

Within the same study, an animal model of celiac disease was developed. Intestinal villous atrophy was induced in male Wistar rats with a single dose of methotrexate. Rats with induced intestinal inflammation had elevated levels of endocannabinoids in the jejunum. Anandamide, 2-AG, and PEA levels peaked on day three of treatment in the muscle/serosa layer and returned to the basal values on the seventh day alongside recovery. In mucosal samples, the levels of PEA and 2-AG were significantly higher than those in the control group, while the concentration of anandamide did not differ significantly [104].

An experiment conducted on human explant colitis models suggested that CB2 receptor activation may reduce cytokine-evoked mucosal damage in the intestines. The results of the study showed that anandamide reduced induced tissue destruction and lowered the number of lymphocytes in the mucosa of human explants. Moreover, JTE-907, the CB2 receptor inverse agonist, caused loss of epithelial integrity, which strongly suggested that it was through this receptor that the protective effects of anandamide had occurred. However, not all endocannabinoid impact was completely reversed during CB2R blockade, suggesting an alternative route of tissue protection. Further testing with a target-specific CB1R agonist did not have the expected effect, and the expression of the receptor itself in the human tissues used in this study was found to be relatively low; the role of other receptors or mechanisms should be therefore suspected and examined [96]. The same study examined the impact of anandamide, CBD, and the CB1 and CB2 receptor agonists ACEA and JWH-015 on Caco-2 monolayer permeability, finding no significant effects on TEER. The substances also did not reverse the fall in TEER caused by cytokine-induced damage to the Caco-2 cells [96]. 

Endocannabinoids have a high potential for repairing cytokine-evoked damage in the case of colitis and, most likely, other inflammation within the GIT. As stated before, the mechanism itself has not been fully investigated, but it is certain that the range of action and possibilities of these substances is quite wide.

### 6.2. Therapeutic Potential of Phytocannabinoids

There has been little clinical research investigating the effects of cannabinoids on the GIT physiology as well as on pathological states (Table 6). A particularly important source of reliable data in this area is clinical trials, the analysis of which indicates a number of limitations and even inconsistent results. However, there are studies using animal and tissue models suggesting that they may be useful in managing a variety of GIT diseases, including *Clostridium difficile*-associated colitis, IBS, and gastro-esophageal reflux.

#### 6.2.1. Therapeutic Potential of THC and CBD

##### Infectious Diseases

CBD has been shown to dose-dependently restore cell viability, TEER values, ZO-1, and occludin expression in Caco-2 cell cultures treated with TcdA. CBD also reversed the fall in RhoA GTP (the primary target for TcdA) expression and reversed the increase in expression of the pro-apoptotic Bax protein. All these protective effects were abolished by the CB1 antagonist AM251. This suggests that CBD may be helpful in clinical management of *Clostridium difficile*-associated colitis [95]. THC upregulated PPARγ mRNA expression (which was reduced in SIV-infected individuals) when incubated ex vivo with intact colonic tissue of chronically SIV-infected rhesus macaques [94].

In mice with LPS-induced sepsis, CBD further reduced GIT motility, worsening septic ileus. This was prevented by the CB1 receptor antagonist AM251. CBD had no effect on GIT motility in healthy control mice. LPS also significantly upregulated CB1 receptor expression in the small intestine without affecting CB2 receptor expression. CBD reversed LPS-induced upregulation of FAAH activity, an effect that was unmodified by CB1 blockade. This suggests that CBD’s exacerbation of septic ileus is due to FAAH inhibition and imbalance between endocannabinoid production and degradation, leading to endocannabinoid-induced inhibition of motility via CB1 receptor activation [105]. 

##### Therapeutic Potential of THC and CBD in Gastrointestinal Tract Motility

In an in vivo study, THC was observed to dose-dependently decrease the frequency of transient lower esophageal sphincter relaxation episodes (which are the main mechanism of gastro-esophageal reflux) in both dogs and humans. Additionally, THC caused a dose-dependent decrease in postprandial acid reflux rates in dogs and a non-significant decrease in acid reflux episodes in humans. The CB1 receptor antagonist SR141716A reversed the effect of THC on transient lower esophageal sphincter relaxations (TLESR) [106]. A 4-week randomized controlled trial investigating the effect of CBD on clinical signs of diabetic or idiopathic gastroparesis showed improvements in symptom severity and frequency in the CBD group. CBD also caused delayed gastric emptying of solids in patients, but provided good clinical relief of symptoms despite this [107]. On the other hand, CBD provided no significant amelioration of symptoms in patients suffering from functional dyspepsia without delayed gastric emptying [108]. Similarly to CBD, THC also delayed gastric emptying in healthy subjects, as well as increasing fasting gastric volume in males. Colonic transit, however, was not affected [109]. In contrast, a later study documented a reduction in postprandial colonic motility and tone in response to THC in healthy subjects, alongside an increase in colonic sensitivity to distension [110]. Another possible interaction of cannabis use with gastric emptying was established in an epidemiological study which linked cannabis use to hospitalization with gastroparesis. This suggests that the delaying effect of cannabis on gastric emptying may cause dysmotility when used in excess [111].

There are case studies of THC being successfully used in clinical practice. One such example is the almost complete remission of symptoms related to failure to thrive secondary to intestinal dysmotility in a 58-year-old patient. When treated with dronabinol, the patient was able to maintain her nutritional status orally, which had previously been impossible [112]. Another such case is described for a 19-year-old patient suffering from chronic intestinal pseudo-obstruction, who had been dependent on parenteral nutrition since birth. After commencement of treatment with dronabinol, she experienced marked improvement in symptoms and subjective quality of life [113]. 

##### Therapeutic Potential of THC and CBD in Inflammatory Diseases

In a murine model of DSS-induced colitis, CBD co-administered with fish oil at doses found to have no effect individually was able to effect several beneficial changes. CBD at 1 mg/kg in combination with fish oil dosed at 20 mg/kg was able to reduce disease activity index, colonic weight/length ratio, and tissue myeloperoxidase activity and ameliorated the DSS-induced rise in intestinal permeability [98]. A similar model using DNBS as the colonic irritant found no protective effect for CBD as a sole agent, but as in the previously described study, fish oil potentiated CBD, such as to allow it to attenuate colonic inflammation, as measured by a reduction in colon weight/length ratio, myeloperoxidase activity, colonic interleukin 1β levels, and intestinal permeability [99].

In a trial conducted in patients suffering from ulcerative colitis, THC administration via smoking of THC-rich cannabis caused circulating endocannabinoid levels to be maintained throughout the study, as opposed to the gradual decrease noted in the placebo group. Serum 2-AG was correlated positively with quality of life score, and serum anandamide, PEA, and OEA levels were negatively correlated with bowel movement frequency. At the end of the trial, the THC group had improved quality of life scores and disease symptoms relative to placebo, presumably via altering endocannabinoid tone. The mechanism by which THC might raise circulating endocannabinoid levels is not currently known [100]. Within the same study, serum endocannabinoids continued to fall in patients suffering from Crohn’s disease and did not respond to an orally dosed mixture of THC and CBD. However, clinical response was noted in both groups [100]. Patients with Crohn’s disease also were able to make significant clinical improvements (including complete remission and weaning from steroid dependency) when enrolled in a clinical trial, in which subjects smoked THC-rich cannabis cigarettes [114]. 

CBD-containing chewing gum was not shown to improve pain or quality of life scores in a randomized controlled trial conducted in patients suffering from IBS [115], and was equally unsuccessful in affecting disease activity in patients suffering from Crohn’s disease [116]. Data from a trial investigating the therapeutic effect of CBD on ulcerative colitis suggest that CBD may be beneficial for symptomatic treatment. However, compliance was quite low in the CBD group, and the primary endpoint of patients in remission at week 10 was not met [117].

Dronabinol significantly improved colonic compliance and decreased colonic motility in patients with IBS type D and A at 5 mg. Dronabinol did not affect colonic sensation or fasting and postprandial colonic tone [118]. However, a later study found no significant effect on gastric, small bowel, or colonic transit in patients suffering from type D IBS. There was no significant interaction between treatment effect and FAAH genotype in the study [119]. 

#### 6.2.2. Therapeutic Potential of Other Phytocannabinoids

In a murine model of DNBS-induced colitis, CBG reversed colonic weight/length ratio increase and reduced histological signs of colonic injury. In addition, CBG improved DNBS-induced intestinal barrier disruption, as shown by it abolishing the rise in serum penetration of FITC-conjugated dextran. CBG lowered inflammatory markers such as MPO activity and iNOS expression (but not COX-2) and normalized colonic IL-1b, IL-10, and IFNg levels. In the same study, CBG restored decreased SOD activity and limited ROS production [120]. This suggests CBG may be helpful in the treatment of IBS. In a similar model of murine colitis, also induced by DNBS, CBG was able to reduce tissue myeloperoxidase activity, colonic interleukin 1β levels, and intestinal permeability [99].

One study found that CBN delayed GIT transit in vivo in healthy mice but was more effective in mice with intestinal inflammation induced by croton oil. This effect was mediated via CB1 receptors, as it was blocked by the CB1 receptor antagonist rimonabant. This study also showed upregulation of CB1 receptor expression in the inflamed intestine, as well as increased FAAH activity in the intestinal lumen [60].

CBDV given i.p. and p.o. was found to reduce serum levels of orally administered FITC-dextran in mice with DNBS-induced experimental colitis, suggesting a decrease in inflammatory GIT hyperpermeability [121].

#### 6.2.3. Summary of Phytocannabinoid Clinical Trials

The clinical trials listed in the Table 6 are selected from trials listed as completed in the Cochrane Library, NIH clinicaltrials.gov, and PubMed, with published results. Trials older than 20 years were excluded, as well as studies which were not placebo-controlled. 

There are several noteworthy limitations in this literature. The complete number of trials is not large, and some studies are quite old. Most of the studies operated on small cohorts, presumably due to the difficulty of recruiting large numbers of patients willing and able to participate in a clinical trial. There is only one available trial investigating the effect of cannabinoids on intestinal permeability. Some studies were complicated by patient compliance issues, where subjects found the dosage or route of administration troublesome [115,117]. In studies where THC was administered via cannabis smoke inhalation, it is difficult to isolate the effect of THC from other trace phytocannabinoids [100,114]. Similarly with administration of oil containing a mixture of THC and CBD, ascribing a particular effect to one substance or the other is challenging [100]. The dosage of CBD used in these clinical trials varies widely, from 20 mg per day [116] to around 1400 mg per day [108]. Some trials using similar phytocannabinoid dosages have seemingly incompatible results; one study reported no change in colonic transit [109], while the other reported reduced colonic motility [110]. These studies do not lend themselves to comparison due to different routes of administration, mixtures of phytocannabinoids, and variation in dosage. 

Thus, more preclinical studies are needed to establish a mechanistic basis of cannabinoid activity, allowing future clinical trials to use more standardized protocols. 

The list of clinical trials investigating the influence of cannabinoids on the GIT is given in Table 6.

**Table 6:** Clinical trials investigating the influence of cannabinoids on the GIT.

Phytocannabinoid	Disease Status and Number of Subjects	Cannabinoid Dose	Study Type	Effect	Year	Reference
THC	Healthy: 30	7.5 mg; 5 mg b.i.d.	double-blinded, randomized, placebo-controlled, parallel-group study	Slowing of gastric emptying Greater fasting gastric volume in males No effect on colonic transit	2006	[109]
THC	Healthy: 52	7.5 mg	double-blinded, randomized, placebo-controlled, parallel-group study	Colonic relaxation Reduction in postprandial colonic motility and tone Increase in sensitivity to colonic distension	2007	[110]
CBD, PEA	Healthy: 30	CBD: 600 mg PEA: 600 mg	double-blinded, randomized, placebo-controlled, parallel-group study	CBD and PEA lowered urinary lactulose-to-mannitol ratio, implying decrease in intestinal permeability	2019	[92]
THC	IBS-C: 35 IBS-D: 35 IBS-A: 5	2.5 mg, 5 mg	double-blinded, randomized, placebo-controlled, parallel-group study	Increased colonic compliance Decreased fasting proximal and distal left colonic motility index Effects were greatest in IBS-D or A patients	2011	[118]
THC	IBS-D: 36	2.5 mg; 5 mg b.i.d.	double-blinded, randomized, placebo-controlled, parallel-group study	No significant effects on gastric, small bowel, or colonic transit No significant interaction between treatment effect and FAAH genotype	2012	[119]
CBD	IBS: 32	50 mg (ad libitum)	double-blinded, randomized, placebo-controlled, cross-over trial	No significant effects of CBD chewing gum on pain scores	2022	[115]
THC (in cannabis flowers)	Crohn’s Disease: 21	11.5 mg per cigarette	double-blinded, randomized, placebo-controlled, parallel-group study	5 out of 11 patients achieved complete remission 10 out of 11 patients achieved clinical response (decease in CDAI score) 3 out of 11 patients were weaned from steroid dependency	2013	[114]
CBD	Crohn’s disease: 21	10 mg b.i.d.	double-blinded, randomized, placebo-controlled, parallel-group study	No effect on disease activity	2013	[116]
THC, CBD	Crohn’s disease: 30 Ulcerative colitis: 19	2 mg/mL THC and 8 mg/mL CBD in oil, up to 1 mL b.i.d. (Crohn’s disease) 11.5 mg THC per cigarette (ulcerative colitis)	double-blinded, randomized, placebo-controlled, parallel-group study	THC prevented decrease in circulating endocannabinoids in ulcerative colitis patients Higher circulating endocannabinoid levels were associated with fewer bowel movements and higher quality of life Clinical response was noted in both ulcerative colitis and Crohn’s disease patients	2021	[100]
CBD	Ulcerative Colitis: 60	250 mg b.i.d.	double-blinded, randomized, placebo-controlled, parallel-group study	The trial did not meet its primary endpoint However, data suggests symptomatic improvement	2015	[117]
CBD	Functional dyspepsia with normal gastric emptying: 48	10 mg/kg b.i.d.	double-blinded, randomized, placebo-controlled, parallel-group study	CBD did not significantly alter satiation, gastric motor function, or symptoms following food ingestion CBD did not delay gastric emptying Neither CNR1 or FAAH genotype variation significantly influenced treatment response	2022	[108]

#### 6.2.4. Phytocannabinoid Adverse Effects

The most common adverse effects associated with cannabis use are vomiting and abdominal pain. A subset of users may also develop cannabinoid hyperemesis syndrome (CHS), which is described in individuals chronically engaging in heavy cannabis use. Those affected by CHS experience abdominal pain and persistent vomiting, which is often improved by compulsive hot showers. Symptoms of CHS cease upon termination of cannabis use [122,123,124,125]. Another serious condition associated with cannabis use is adult intussusception (AI). Intussusception refers to invagination of one bowel segment onto another, potentially leading to obstruction or bowel perforation if not treated. This condition mostly occurs in children, and in adults is usually secondary to another disease, such as neoplasia or post-operative adhesions or strictures. No underlying functional causes were found in individuals suffering from cannabis-associated AI. Patients complained of severe abdominal pain, vomiting, diarrhea, and abdominal cramps [124]. 

Symptoms are dependent on mode and frequency of use, with inhalation of cannabis being a risk factor for developing chronic bronchitis and exacerbating pre-existing respiratory conditions. Oral administration of THC is not burdened with the above adverse effects, but is characterized by much lower systemic bioavailability, as well as slow and highly variable absorption [126]. Frequent cannabis use is associated with greater risk of developing cannabis use disorder (CUD), as well as possible negative effects on cognition [125]. 

Adverse effects associated with CBD administered orally were less severe, the most common being gastrointestinal symptoms such as diarrhea, vomiting, nausea, abdominal pain, and constipation. The most common non-GI related adverse effects were somnolence, fatigue, and elevation of serum ALT/AST activity [127].

It is important to bear in mind that studies investigating adverse effects of cannabinoids are few in number, and often have few participants. Some adverse effects such as CHS and cannabis-associated AI are described only in case studies, and the physiological mechanisms by which they occur are far from obvious. This strongly shows the need for more studies investigating the mechanistic basis of cannabinoid effects on the GIT, as well as interactions between cannabinoids and other substances affecting the GIT. The lack of data on adverse effects for endocannabinoids is probably due to their rapid breakdown by FAAH and MAGL in vivo.

## 7. Conclusions

In summary, our narrative review highlights the complex interaction between cannabinoids and gastrointestinal physiology, shedding light on their potential therapeutic applications in the treatment of GIT diseases. The findings highlight the diverse effects of cannabinoids on motility, intestinal permeability, and inflammation, which are mediated by interactions with endocannabinoids and cannabinoid receptors. It is noteworthy that cannabinoids such as THC and CBD exhibit receptor-specific effects on GIT motility via CB1 receptors, causing inhibition of muscle contractility, which may suggest targets for therapeutic interventions. Moreover, the involvement of CB1 and CB2 receptors in regulating intestinal permeability underscores the complexity of mechanisms mediated by cannabinoids in gastrointestinal health. In addition, cannabinoids show promise as anti-inflammatory agents, offering potential benefits in the treatment of Crohn’s disease, ulcerative colitis, and IBD. Moreover, their role in modulating intestinal motility and relieving pain implicates cannabinoids as potential agents for improving quality of life in gastrointestinal disorders, especially chronic such as IBS. The results of clinical trials and data on the adverse effects of phytocannabinoids indicate that further research is needed to elucidate the exact mechanisms and optimize therapeutic strategies to realize the full potential of cannabinoids in clinical practice. 

## 8. Literature Search Methodology

To conduct the literature review, major scientific databases such as PubMed, Scopus, and Web of Science were searched using appropriately selected keywords and specific search criteria, such as (can-nabinoids OR phytocannabinoids OR cannabinoid OR phytocannabinoid OR THC OR CBD OR CBN OR CBC OR CBG OR THCA OR CBDA OR CBGA OR tetrahydrocanna-binol OR cannabidiol OR cannabinol OR cannabichromene OR cannabigerol OR tetrahy-drocannabinolic acid OR cannabidiolic acid OR cannabigerolic acid) AND ((gastrointestinal OR GI OR GIT OR intestinal OR gut OR gastric) AND (motility OR passage OR paresis OR stasis OR peristalsis OR propulsion OR contractility) OR (permeability OR TEER OR barrier OR FD4)). The selection of articles was limited to those thematically related to the subject under discussion. In addition, citation lists of selected articles were reviewed to identify additional potential publications for inclusion in the analysis.

## Figures and Tables

**Table 1:** Properties of selected phytocannabinoids.

Phytocan-Nabinoid	CB1 Agoni-Zation	CB1 Antago-Nization	CB2 Agonization	CB2 Antago-Nization	Additional Mechanisms	References
THC	+	−	+	−	voltage-dependent N and P/Q-type calcium channel inhibitor GPR55, GPR18 agonist antagonizing of serotonin 5-HT3a receptors PPARγ agonist TRPA1, TRPV2, 3, and 4 agonist positive allosteric modulator of the m- and d-opioid receptors and glycine receptors, subunits a1 and a3	[42,44]
CBD	−	Very weak	−	Very weak	CB1 receptor allosteric modulator GPR55 antagonist FAAH inhibitor anandamide reuptake inhibitor positive allosteric modulator at a1 and a1b glycine receptors positive allosteric modulator at m- and d-opioid receptors TRPA1, TRPV1, and TRPV2 agonist TRPM8 antagonist competitive adenosine uptake inhibitor PPARγ agonist serotonin receptor 5-HT1a agonist intracellular calcium ion regulator T-type calcium ion channel inhibitor suppressing of tryptophan degradation 5-lipoxygenase and 15-lipoxygenase inhibitor phospholipase A2 stimulator/inhibitor antioxidant	[4,45,46]
CBG	weak	−	partial	−	TRPA1 agonist TRPV1-4 agonist TRPM8 antagonist Alpha-2 adrenergic agonist 5-HT1a antagonist PPARγ agonist	[41,47]
CBN	partial		partial		TRPV2 agonist TRPA1 agonist	[42,47]
CBC	−	−	−	−	TRPV1 agonist TRPA1 agonist antibiotic, antiviral, and antifungal properties	[4]
CBDV	−	−	−	−	TRPV4 agonist DAGL-alpha inhibitor	[47,48]
THCV	−	+	partial	−		[43]
Cannabinoid acids	−	−	−	−	TRPA1 partial agonists TRPM8 antagonists COX-2 inhibitor	[43]

GPR55, GPR18: G protein-coupled receptors 55 and 18; PPARγ: peroxisome proliferator-activated receptor gamma; TRPA1: transient receptor potential cation channel subfamily A member 1; TRPV1, 2, 3, and 4: transient receptor potential cation channel subfamily V member 1, 2, 3, and 4; TRPM8: transient receptor potential cation channel subfamily M member 8; FAAH: fatty acid amide hydrolase; DAGL: diacylglycerol lipase.

**Table 2:** Summary of endocannabinoid influence on gastrointestinal motility.

Substance	Species	Experiment Type	GIT Segment	Effect	Mechanism	References
Anandamide	Mouse	Ex vivo	Ileal longitudinal muscle strips	Reduction in spontaneous contractility	CB1	[53]
Anandamide	Mouse	Ex vivo	Colon	Reduction in electrically evoked contractions	CB1	[54]
Anandamide	Mouse	In vivo	Stomach and small intestine	Reduction in GIT motility Improvement in postprandial glycemia	n/d	[58]
Anandamide	Guinea pig	Ex vivo	Small intestine circular muscle preparations	Reduction in cholinergic and NANC contractility	CB1	[55]
Anandamide, 2-AG	Human	Ex vivo	Colonic longitudinal and circular muscle preparations	Reduction in contractile response to ACh	Non-CB1, non- CB2 pathway	[56]
Anandamide	Rat	Ex vivo	Ileal preparations	Reduction in contractile response to ACh	CB1	[57]
Anandamide	Guinea pig	Ex vivo	Proximal small intestine myenteric plexus longitudinal muscle strips	Increase in basal ACh release and muscle tone	TRPV1	[50]
Anandamide	Guinea pig	Ex vivo	Proximal small intestine myenteric plexus longitudinal muscle strips	Inhibition of electrically evoked ACh release and contractions	n/d	[50]
Anandamide, 2-AG	Guinea pig	Ex vivo	Distal colon longitudinal muscle strips	Increase in contraction	Possible involvement of lipoxygenase metabolites	[52]
Anandamide	Guinea pig	Ex vivo	Ileum	Increase in contraction	Possible involvement of cyclooxygenase metabolites	[59]
Anandamide	Mouse	In vivo	Small intestine	Possible causative role in paralytic ileus	CB1	[62]
Anandamide	Mouse	In vivo	Small intestine	Decrease in anandamide levels linked to increased GIT transit	n/d	[63]
Anandamide	Guinea Pig	Ex vivo	Small intestine myenteric plexus longitudinal muscle strips	Inhibition of electrically induced contraction Low dose: inhibition of ethylenediamine-induced GABA release High dose: Enhancement of relaxation caused by ethylenediamine	CB1 CB1 TRPV1	[64]
Anandamide	Guinea Pig	Ex vivo	Small intestine myenteric plexus longitudinal muscle strips	Inhibition of electrically induced adenosine release	CB1	[65]
Anandamide	Rat	Ex vivo	Colon	Inhibition of excitatory cholinergic and inhibitory VIPergic motor neurons Inhibition of sensory neurons	CB1	[66]
PEA	Mouse	In vivo	Small intestine	Inhibition of GI transit	n/d	[67]
OEA	Mouse	In vivo	Small intestine	Inhibition of GI transit Inhibition of stress-induced hypermotility	n/d	[67,69]
OEA	Mouse	In vivo	Stomach	Inhibition of gastric emptying	n/d	[70]
OA	Mouse	In vivo	Small intestine	Inhibition of GI transit	n/d	[67]

CB1—cannabinoid receptor type 1; CB2—cannabinoid receptor type 2; TRPV1—transient receptor potential cation channel subfamily V member 1; n/d—not determined.

**Table 3:** Summary of phytocannabinoid influence on gastrointestinal motility.

Substance	Species	Experiment Type	GIT Segment	Effect	Mechanism	References
THC	Mouse	In vivo	Small intestine	Inhibition of GI transit	n/d	[72,73,77]
THC	Guinea Pig	Ex vivo	Ileum	Inhibition of contractile response to electrical and 5-HT stimulation	n/d	[74]
THC	Guinea Pig	Ex vivo	Small intestine myenteric plexus longitudinal muscle strips	Inhibition of contractile response to electrical stimulation	CB1	[75,76]
THC	Rat	Ex vivo	Small intestine myenteric plexus longitudinal muscle strips	Inhibition of contractile response to electrical stimulation	CB1	[78]
CBD	Mouse	In vivo	Small intestine	Normalization of inflammatory hypermotility	CB1	[79]
CBD	Mouse	Ex vivo	Ileum	Inhibition of ACh-induced contractions	CB1	[79]
CBD	Rat	Ex vivo	Colon	Inhibition of ACh-induced contraction Synergistic inhibition of ACh-induced contraction with diclofenac	n/d	[81]
CBD	Rat	Ex vivo, in vivo	Upper GIT, colon	Normalization of inflammatory hypermotility	n/d	[82]
CBD, CBDA	Asian house shrew	Ex vivo	Proximal and distal intestinal segments	Lowering of resting tension of segments inhibition of carbachol-induced contractions relaxation of KCL-contracted segments	n/d	[80]
CBC	Mouse	In vivo	Small intestine	Normalization of inflammatory hypermotility	n/d	[61]
CBC	Mouse	Ex vivo	Ileum	Inhibition of contractile response to electrical stimulation and ACh	N and L-type Ca^2+^ channels	[61]
CBN	Guinea Pig	Ex vivo	Small intestine myenteric plexus longitudinal muscle strips	Inhibition of contractile response to electrical stimulation	CB1	[76]
CBN	Mouse	In vivo	Colon	Inhibition of colonic propulsion	CB1	[83]

n/d—not determined; CB1—cannabinoid receptor type 1.

**Table 4:** Summary of endocannabinoid influence on intestinal permeability.

Substance	Species/Cell Culture	Experiment Type	Effect	Mechanism	References
anandamide	Caco-2	In vitro	Apical: increase in permeability Basolateral: decrease in permeability Claudin-1 mRNA downregulation	CB1 CB1, TRPV1 CB1	[86]
2-AG	Caco-2	In vitro	Apical: increase in permeability Basolateral: decrease in permeability Claudin-1 mRNA downregulation	CB1 CB1 CB1	[86]
Anandamide, 2-AG	Caco-2	In vitro	Apical: increase in permeability Stronger increase in the presence of FAAH/MAGL inhibitors	CB1 CB2	[86]
Anandamide	Caco-2	In vitro	Apical: increase in permeability Basolateral: decrease in permeability	CB1 CB1, TRPV1	[88]
2-AG	Caco-2	In vitro	Apical and basolateral: increase in permeability	CB1	[88]
OEA	Caco-2	In vitro	Apical: decrease in permeability Basolateral: increase in permeability Reduction in membrane expression of AQP3 and AQP4	TRPV1 PPARα n/d	[90,91]
PEA	Caco-2	In vitro	Apical and basolateral: decrease in permeability Reduction in membrane expression of AQP3 and AQP4	PPARα n/d	[90]
NADA, OA	Caco-2	In vitro	Apical: decrease in permeability Basolateral: decrease in permeability	TRPV1 TRPV1, CB1 (OA)	[91]
NE, PEA	Caco-2	In vitro	Basolateral: decrease in permeability	PPARα	[91]
PEA	Caco-2	In vitro	Basolateral: decrease in permeability Prevention of cytokine-induced drop in TRPV1 mRNA, and rise in AQP3 gene expression Increase in AQP4 gene expression	PPARα, PKA, MAPK, and adenylyl cyclase signaling pathways	[92]
PEA	Human	Ex vivo	Decrease in claudin-3 mRNA	n/d	[92]
PEA	Human	In vivo	Lowering of urinary lactulose-to-mannitol ratio, implying decrease in intestinal permeability	n/d	[92]

CB1—cannabinoid receptor 1; CB2—cannabinoid receptor 2; TRPV1—transient receptor potential cation channel subfamily V member 1; PPARα—peroxisome proliferator-activated receptor gamma; PKA—protein kinase A; MAPK—mitogen-activated protein kinase; n/d—not determined.

**Table 5:** Summary of phytocannabinoid influence on intestinal permeability.

Substance	Species/Cell Culture	Experiment Type	GIT Segment	Effect	Mechanism	Reference
THC, CBD	Caco-2	In vitro	n/a	Decrease in permeability Upregulation of ZO-1 mRNA	CB1 CB1	[86]
THC, CBD	Caco-2	In vitro	n/a	Apical: decrease in permeability	CB1	[87]
CBD	Caco-2	In vitro	n/a	Apical: decrease in permeability	CB1	[93]
THC	Caco-2	In vitro	n/a	Decrease in permeability	PPARγ upregulation	[94]
THC	Rhesus macaque	Ex vivo	Colon	Decrease in permeability	PPARγ upregulation	[94]
CBD	Caco-2	In vitro	n/a	Reversal of decreased gene expression for ZO-1 and occludin caused by TcdA	CB1	[95]
CBD	Caco-2	In vitro	n/a	Apical: decrease in permeability Prevention of cytokine-induced drop in TRPV1 mRNA, and rise in AQP3 gene expression	CB1, PKA, MAPK, and adenylyl cyclase signaling pathways	[92]
CBD	Human	Ex vivo	Colonic mucosa and submucosa	Inhibition of inflammation-induced decrease in claudin-5 mRNA	n/d	[92]
CBD	Human	In vivo	n/a	Lowering of urinary lactulose-to-mannitol ratio, implying decrease in intestinal permeability	n/d	[92]
CBD	Mouse	In vivo	n/a	No effect on inflammation-induced hyperpermeability as sole agent Amelioration of hyperpermeability when co-administered with fish oil	n/d	[98,99]
CBG	Mouse	In vivo	n/a	Amelioration of inflammation-induced hyperpermeability as sole agent Increased effect when co-administered with fish oil	n/d	[98]

ZO-1—cona occludens-1; CB1—cannabinoid receptor 1; PPARα—peroxisome proliferator-activated receptor gamma; TRPV1—transient receptor potential cation channel subfamily V member 1; PKA —protein kinase A; MAPK—mitogen-activated protein kinase; n/d—not determined.
